# Algorithms to Improve the Prediction of Postprandial Insulinaemia in Response to Common Foods

**DOI:** 10.3390/nu8040210

**Published:** 2016-04-08

**Authors:** Kirstine J. Bell, Peter Petocz, Stephen Colagiuri, Jennie C. Brand-Miller

**Affiliations:** 1 Charles Perkins Centre, and the School of Life and Environmental Sciences, the University of Sydney, Sydney 2006, Australia; Kirstine.Bell@sydney.edu.au (K.J.B.); Stephen.Colagiuri@sydney.edu.au (S.C.); 2 Department of Statistics, Macquarie University, Sydney 2109, Australia; peter.petocz@mq.edu.au

**Keywords:** glycaemia, insulin, carbohydrate, protein, fat, glycemic index, food insulin index

## Abstract

Dietary patterns that induce excessive insulin secretion may contribute to worsening insulin resistance and beta-cell dysfunction. Our aim was to generate mathematical algorithms to improve the prediction of postprandial glycaemia and insulinaemia for foods of known nutrient composition, glycemic index (GI) and glycemic load (GL). We used an expanded database of food insulin index (FII) values generated by testing 1000 kJ portions of 147 common foods relative to a reference food in lean, young, healthy volunteers. Simple and multiple linear regression analyses were applied to validate previously generated equations for predicting insulinaemia, and develop improved predictive models. Large differences in insulinaemic responses within and between food groups were evident. GL, GI and available carbohydrate content were the strongest predictors of the FII, explaining 55%, 51% and 47% of variation respectively. Fat, protein and sugar were significant but relatively weak predictors, accounting for only 31%, 7% and 13% of the variation respectively. Nutritional composition alone explained only 50% of variability. The best algorithm included a measure of glycemic response, sugar and protein content and explained 78% of variation. Knowledge of the GI or glycaemic response to 1000 kJ portions together with nutrient composition therefore provides a good approximation for ranking of foods according to their “insulin demand”.

## 1. Introduction

Metabolic health underlies the prevention and management of chronic diseases such as type 2 diabetes, cardiovascular disease and some cancers. Dietary patterns which induce excessive insulin secretion are believed to contribute to worsening insulin resistance and beta-cell dysfunction [1,2].

Carbohydrate has been identified as the sole macronutrient that directly increases postprandial blood glucose levels and thus the main dietary determinant of postprandial insulin secretion. However, numerous studies in healthy subjects have demonstrated that the same amount of carbohydrate from different food sources produces wide variations in glycaemic and insulin responses [3,4,5,6]. Furthermore, the insulinotropic effects of protein and fat are now well documented [7,8,9,10] and are comparable to that of carbohydrate in some cases [11,12,13,14,15,16]. In type 1 and type 2 diabetes, fat and protein can significantly impact postprandial glucose excursions in the absence of sufficient insulin [17,18,19,20]. These findings have important implications for the prevention and management of people with diabetes. A better understanding of the relationship between dietary factors and physiological insulin secretion evoked by different diets can also inform nutritional epidemiology.

In order to systematically quantify postprandial insulin responses for a broad range of common foods, a Food Insulin Index (FII) has been proposed which ranks foods based on the insulin response (“demand”) in healthy subjects relative to an isoenergetic reference food [15]. Using food energy as the constant allows all foods to be included, not just those with sufficient carbohydrate content, and thus all dietary components and their metabolic interactions can be considered, allowing a more holistic approach to determining insulin “demand”. While the FII is determined in healthy subjects, our previous studies suggest that the FII may be relevant to predicting exogenous prandial insulin doses in type 1 diabetes [21,22] and reducing postprandial insulinaemia in type 2 diabetes [23]. Although predictive equations have been published, these were developed on the basis of only 38 foods that had been tested at the time, and their accuracy has not been validated [24]. Since 1995, a further 109 common foods in the western diet have been tested for their FII using the same methodology.

Hence, the aim of the present study was to evaluate the degree of association between different dietary factors and the postprandial physiological glycaemic and insulinaemic responses in healthy subjects and validate previously generated regression equations for predicting the FII of a food. Using the larger database, we developed new predictive models to allow improved estimates of dietary “insulin demand”.

## 2. Materials and Methods

### 2.1. FII Testing

The FII testing methodology has been described in detail elsewhere [15]. Briefly, the FII for each subject was determined as the iAUC of the insulin response elicited by the 1000 kJ portion of the test food expressed as a percentage of the average iAUC response to the 1000 kJ portion of the reference food (glucose). The final FII of a food was calculated as the average FII in 10 subjects. The FII for 121 foods have been published [15] and an additional 26 foods have been included in the Online Supplementary Material (Table S1).

### 2.2. Statistical Analyses

To determine the degree of association between different dietary factors and the postprandial physiological glycaemic and insulinaemic responses, linear regression analysis was used to test associations between FII and available carbohydrate, protein, fat, sugar, fibre, glucose score (GS = relative glycaemic response to 1000 kJ food portion), Glycemic Index (GI) and Glycemic Load (GL) for 147 single foods. A subanalysis was also used to test associations between FII and types of fat and select amino acids for those foods with available information. Relationships between macronutrients and FII were also visualised as colour-coded response profiles using thin-plate spline procedures in R (version 3.0.2, The R Foundation, Vienna, Austria). The interactions between carbohydrate, protein and fat on insulin secretion are complex, but representing the relationships graphically in three dimensions allows the relationships to be more readily appreciated.

To validate the previously published FII stepwise linear regression equations [24], individual data from a nested study of 26 foods single foods were calculated using these equations and compared with the 254 actual observations. Correlations between the calculated FIIs and between the calculated and observed FII for the original 446 observations and the new 254 observations were analysed and compared. The combined 700 individual observations were used to generate a new stepwise regression equation to examine the extent to which the different predictors accounted for the variability of the observed postprandial responses. Throughout the paper, the terms “insulin response”, “relative insulin response”, “FII”, “insulin demand” and “insulinotropic ability” essentially refer to the same phenomenon, *i.e*., the degree of postprandial insulinemia in response to ingestion of different foods.

All statistical analyses were carried out by using the SPSS statistical package version 21 (SPSS Inc., Chicago, IL, USA). Differences and correlation coefficients were considered statistically significant if the *p* value was <0.05 and was highly significant if the *p* value was <0.01 (2-tailed).

## 3. Results

The GS and FII values of the 147 foods varied over a wide range, with large variations within and between food groups. GL was the strongest predictor of glycaemic variation, explaining 80% of the variation in the GS. GL was followed closely by GI, which explained 71%, while available carbohydrate explained 66% of the variation (*p* < 0.0001 for all). The relationships between GS and protein and fat were inverse and highly significant (*r* = −0.45 and −0.61 respectively, *p* < 0.001 for both). Both sugar and fibre showed weak, positive associations with GS, but only sugar was a significant predictor of the relative insulin response (*r* = 0.28, *p* = 0.001 for sugar and *r* = −0.12, *p* = 0.135 for fibre).

Individually, GI explained 51% and available carbohydrate explained 47% of the variation in the insulin responses, while their mathematical product, GL, was the strongest individual predictor of the FII, explaining 55% of the variation in FII (*p* < 0.0001 for all). Sugar was also moderately and significantly correlated with the FII (*r* = 0.36, *p* < 0.0001). The association between the protein content of the food and the FII was relatively weak but significant (*r* = −0.27, *p* = 0.01), while the correlation between fat content and FII was inverse, moderately strong and highly significant (*r* = −0.56, *p* < 0.0001). Fibre was the only nutrient that showed virtually no association with insulin responses (*r* = 0.08, *p* = 0.361).

**Figure 1:**
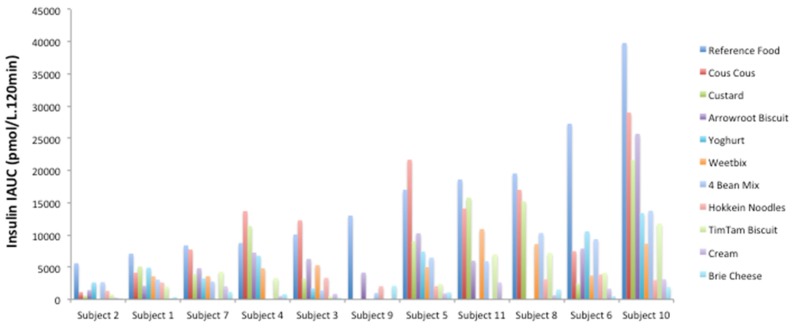
Within-individual insulin responses to 1000 kJ portions of the reference food (glucose) compared to 1000 kJ portions of the test foods over 120 min.

Within individuals, the insulin iAUC to the test foods was generally proportional to the response to the reference food. This is illustrated in a subset of the foods tested by the same group of individuals in Figure 1.

**Figure 2:**
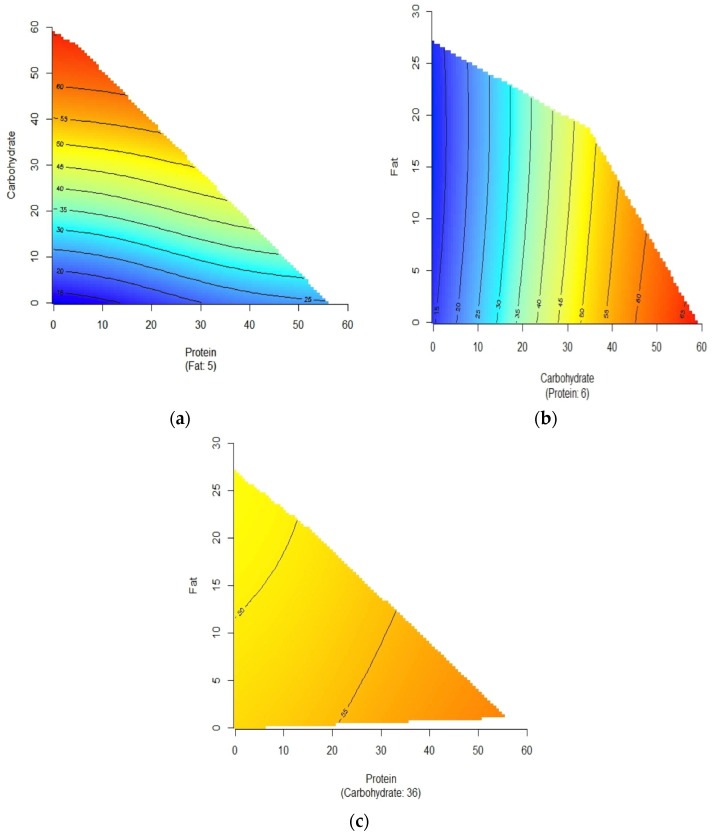
(**a**–**c**) The colour-coded profiles show the relationship between carbohydrate (g/1000 kJ), protein (g/1000 kJ) and fat (g/1000 kJ) and the *Food Insulin Index* (FII) values of common foods. The isolines for FII rise in elevation from dark blue to dark red. For each profile, the third macronutrient is held constant at the median level, that is 5 g of fat/1000 kJ (Figure 2a), 6 g of protein/1000 kJ (Figure 2B) and 36 g of carbohydrate/1000 kJ (Figure 2c).

Carbohydrate, protein and fat contents of each 1000 kJ food portion were mapped against FII as colour-coded response profiles to compare the macronutrients and establish a hierarchy based on their insulinotropic ability (Figure 2). The response profiles visualise the trends in FII as three-dimensional maps, with 2 macronutrients varying on the *X* and *Y* axes (the third macronutrient is held constant at the median level) and the FII values represented by isolines with colour gradation. As expected, the plots show that FII is highest (redder) when carbohydrate is highest and fat or protein are lowest. (Figure 2A,B). When carbohydrate is held constant at the median level, increasing protein has modestly greater effect on FII than increasing fat content (Figure 2C).

**Figure 3:**
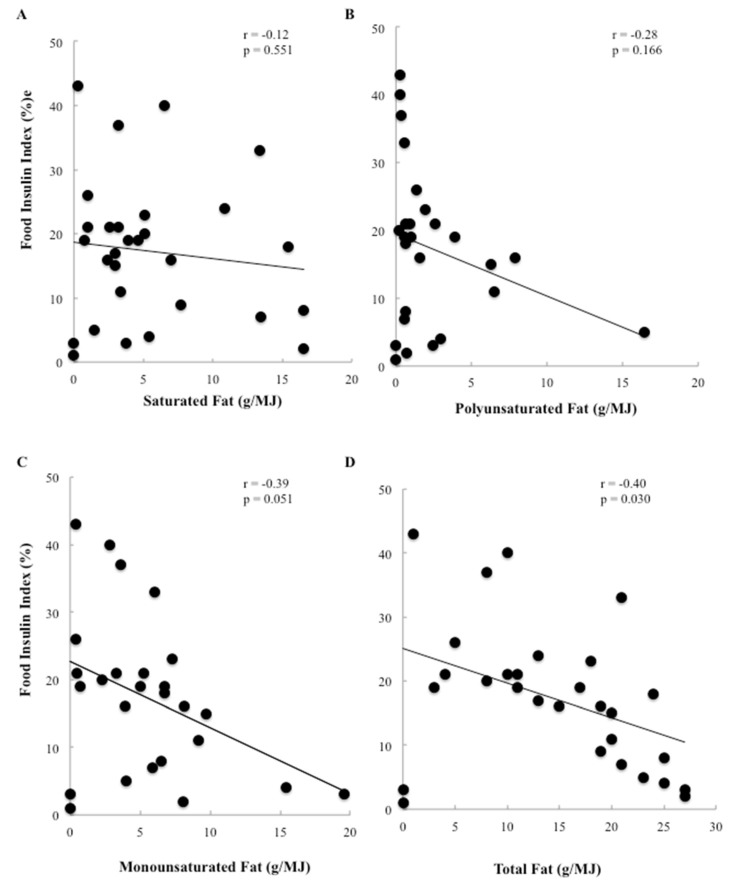
(**A**–**D**) Univariate correlations between the food insulin index and the total fat, saturated fat, polyunsaturated fat and monounsaturated fat for 1000 kJ portions of foods containing 10 g of available carbohydrate or less. **A** & **B**: *n* = 29 single foods; **C** & **D**: *n* = 27 single foods.

To further elucidate whether the type of fat influenced insulin secretion, saturated, polyunsaturated, and monounsaturated fat content per 1000 kJ were correlated with the FII. All 3 types of fat had moderately strong, highly significant inverse associations (*r* = −0.42, −0.37 and −0.51 respectively, *p* < 0.0001 for all. As carbohydrate displaces fat and protein within foods, foods containing <10 g of carbohydrate/1000 kJ were analysed separately (Figure 3). Amongst these 29 low-carbohydrate foods, the relationship between total fat and FII was weakened but remained inverse and significant (*r* = −0.40, *p* = 0.030). However, none of the individual types of fat assessed were significant or strongly correlated.

**Figure 4:**
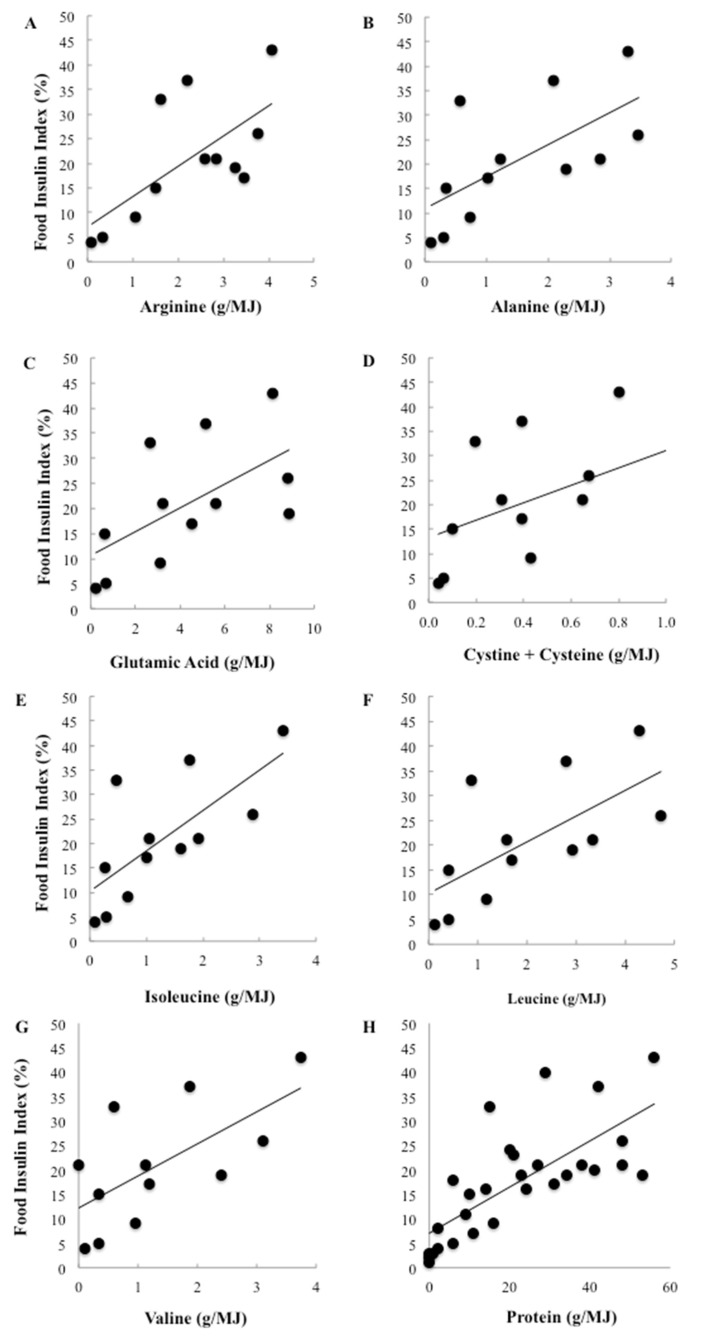
(**A**–**G**) Univariate correlations between the food insulin index and alanine, glutamic acid, arginine, cystine + cysteine, leucine, isoleucine and valine for 1000 kJ portions of single foods containing at least 10 g of available carbohydrate (**A**–**G**: *n* = 12, **H**: *n* = 29).

To further investigate the effect of differing types of proteins, 7 key insulinotropic amino acids were correlated with the FII. Among the 147 foods, the amino acid content could be located for 37 foods [25]. Individual amino acids were only weakly correlated with the observed insulin responses and none were significant. However, when only low carbohydrate foods were included in the analysis, total protein was strongly and positively associated with the FII and highly significant (*r* = 0.73, *p* < 0.0001). Individually, protein accounted for more than half the variation in FII among the low carbohydrate foods (*r*^2^ = 54%). Furthermore, the relationships between the amino acids and the FII became positive and 6 of the 7 identified amino acids were strongly and significantly correlated with relative insulin demand (Figure 4). Of the amino acids, the branched chain amino acids (BCAA) were among the strongest predictors of the FII, with isoleucine accounting for 52%, leucine accounting for 49% and valine accounting for 44% of the variation in insulinaemia (*p* = 0.008, 0.017 and 0.020 respectively). Arginine and alanine were also significant predictors, accounting for 49% and 44% of the variation respectively (*p* = 0.018 and 0.020 respectively).

The previously published prediction equations for the FII can be validated by using the regression equations to calculate the FII for individual observations and comparing with the 254 individual FII observations for the 26 foods in the present study. The correlations were 0.38 and 0.31 respectively for the 2 equations and, although both equations were highly significant (*p* < 0.0001), they were able to account for only 14% and 10% of the variation in the FII respectively. The correlation coefficient between the two equations was 0.95 (*p* < 0.0001).

Given the calculated FII equations were not strong predictors of the observed FII, a new stepwise multiple linear regression analysis of the average FII for the 147 foods was performed (Equation (1)).

(1) FII = − 4.2 + 0.9 GS (%) + 0.3 Sugar (g) + 0.5 Protein (g) + 0.4 Fat (g)

GS, sugar and protein were selected by the model as highly significant predictors of the FII (*p* < 0.001) whereas fat did not reach significance (*p* = 0.053; Table 1) and available carbohydrate, GI and GL were not selected by the model. Together, GS, sugar and protein accounted for 78% of the variation in the observed FII.

Equation (1) selected GS as the most important predictor of FII. However, to obtain a food’s GS, the same methodology for FII testing must be undertaken, and thus the FII could be generated directly if the insulin assay was undertaken. However, unlike glucose assays, insulin analysis is relatively expensive making Equation (1) the next best choice. Alternatively, the GL could be used in place of GS (correlation coefficient between GL and GS, *r* = 0.90). GL can be calculated from the known carbohydrate content and published GI.

Since GL still requires *in vivo* testing in order to determine the prerequisite GI, a second regression equation was developed using only the nutritional composition of the food. Including all significant nutrients in the database, the model selected carbohydrate (*p* < 0.001) and protein (*p* = 0.032) and the equation explained about half of the variation in the FII (*r*^2^ = 49%).

(2) FII = 10.4 + 1.0 Carbohydrate (g) + 0.4 Protein (g)

## 4. Discussion

This study significantly advances our ability to more accurately predict the effect of whole foods and specific dietary factors on postprandial insulinaemia. Using a unique and expanded database, an algorithm that included a measure of the glycaemic impact per MJ (GS), sugar, protein and fat content explained ~80% of the variation in normal postprandial insulinaemia. Known measures of glycaemic impact (GL, GI) and carbohydrate content were the strongest single predictors of the insulinaemia, but explained only 55%, 51% and 47% of variation respectively. Fat, protein and sugar were significant yet relatively weak predictors, accounting for only 31%, 8% and 13% of the variation. At best, macronutrient content (carbohydrate, sugar, fat, protein and fibre per MJ) explained no more than 50% of the variation in insulinaemia, and somewhat surprisingly, fibre content had no significant impact.

These findings indicate that a reliable predictor of likely insulin demand cannot be generated by macronutrient composition alone and that at least one measure of glycaemic impact (GI, GL, GS) is required. Although GS (relative glycemic response per 1000 kJ portion) was superior to GI and GL in this analysis, it is a novel concept that needs further study. The advantage of GS over GI is that virtually any food containing energy, even those low in carbohydrate, can be assessed. In some cases, GS can be extrapolated from a food’s known GI because a 50 g carbohydrate portion has approximately 1000 kJ. However, further studies are needed if GS is to replace GI on a general basis. Clearly, normal physiological insulin secretion is a multifaceted process involving complex nutritional and metabolic interactions between nutrients and the food matrix before and after digestion and absorption.

We also demonstrated large differences in glycaemia and insulinaemia both within and between food groups, inferring that a simple food group approach is not helpful. Despite this intrinsic complexity, our analyses suggest that there is a hierarchy of macronutrients in relation to their potency as insulin secretagogues. Given the strength of the correlations and the colour-coded response profiles, available carbohydrate is easily identifiable as the predominant macronutrient increasing insulin secretion. This is in agreement with the scientific literature and diabetes clinical practice [26,27]. Yet carbohydrate is clearly not the sole macronutrient involved because by itself, it could account for only 47% of the variation in insulinaemia. Indeed, carbohydrate was not selected as a predictor of FII in the multiple regression analysis (Equation (1)). It was only when the model was forced to exclude GS, GL and GI that carbohydrate was selected (Equation (2)).

In this analysis, protein appears to have an inverse relationship with observed insulin responses and is a relatively weak predictor of the FII. This is in direct contrast to the literature, which identifies protein as a potent secretagogue [8]. However, as carbohydrate is such a potent insulin secretagogue, it masks the effects of the other macronutrients. When only the low carbohydrate foods are considered, protein accounts for 53% of the variation, whereas fat accounts for only 16%. This may help explain how high protein foods such as pork, lamb, yoghurt and milk produced notable FII values, in some cases equivalent to their high carbohydrate, low protein counterparts. For example, grilled lamb fillets and hokkien noodles have an almost identical FII of 22 and 21 respectively. In reality, however, a higher protein diet will often be a lower carbohydrate diet. Thus protein tends to displace carbohydrate, the most potent insulin secretagogue.

In contrast, fat had an inverse relationship with FII when low-carbohydrate foods as well as when the entire database were considered. This indicates that a higher fat content lowers the FII under both circumstances. This may be because fat slows gastric emptying and therefore the digestion and absorption of carbohydrate, the primary insulin secretagogue. Fatty acids themselves do not directly stimulate insulin secretion and, in isolation, are relatively weak secretagogues [7]. However, when *added* to a glucose load, they are able to significantly amplify glucose-stimulated insulin secretion beyond that of glucose alone [28]. It is also possible that an extended duration of FII testing (from 2 to 5 h) may reveal a delayed rise in insulinemia. The insulinotropic effect of fat may also be modulated by the type of fat within the food. The literature suggests that the potency of fat on insulin secretion is correlated with the degree of unsaturation, however other studies indicate that the postprandial insulin response is essentially unaffected by the type of fat [29,30]. The latter is consistent with the present findings where we found there were moderately strong, inverse relationships between fat and FII, irrespective of the type of fat.

Our analysis also attempted to elucidate the effect of different types of protein by examining the extent of the associations between the FII and specific insulinotropic amino acids. Of the amino acids assessed, the BCAA, leucine, isoleucine and valine, were the strongest individual predictors of the FII for low carbohydrate foods. This finding is consistent with reports that by themselves these amino acids are potent secretagogues [8,9], via activation of mTOR, which increases insulin secretion [31]. The efficacy of dairy products in stimulating insulin secretion despite their low GI, has been attributed to their high BCAA content. During the cheese manufacturing process, milk is separated into curds and whey, and whey in particular is high in BCAA. This may explain why milk-based products, such as low-fat strawberry yoghurt (FII = 84), vanilla ice-cream (FII = 65) and custard (FII = 57) had a much higher FII than the cheeses, including brie cheese (FII = 7), cheddar cheese (FII = 33) [15] and cream cheese (FII = 18) [15], which have had the whey component, and thus the BCAA, removed.

We also sought to validate the accuracy of the previously published FII prediction equations [24], a practice that is highly recommended yet rarely carried out. The original regression analysis yielded two equations, one including fat but not protein and the other including protein but not fat. The correlations achieved between the calculated FIIs using these equations and the actual observed FII for the 26 foods in the nested study in this paper were lower than that published in the original paper (0.38 and 0.31 *vs.* 0.49 and 0.48), although that is to be expected. Given the calculated FII equations were not strong predictors of the observed FII, a new stepwise multiple linear regression analysis using the average FII for the 147 foods was performed. The new equations explained 77% of the variation in insulinaemia compared with just 32% for both of the original equations.

Our study has several strengths. The FII and GS testing protocol parallels the International Standard of Operation (ISO) for GI testing to ensure reliable data. Because glucose and insulin responses are both highly variable within and between subjects, subjects acted as their own control, thus minimising interindividual differences. Repeat testing of the reference food (tested 3 times) was also applied to reduce intra-individual variation and ensure greater precision. Furthermore, glucose and insulin sampling was done using capillary blood rather than venous blood as incremental glycaemic responses display a greater magnitude and less variability when measured in capillary *vs.* venous blood [26].

The limitations should be noted. The FII is determined by 120 min test sessions as per the GI testing ISO Standard. A 2-h period may be appropriate in some individuals but not others. In healthy subjects but not those with diabetes, the physiologic insulin release will have stabilised postprandial glycaemia and returned it to the baseline level by 120 min and therefore longer test sessions are unnecessary [32]. Potentially, the same is not true for insulin secretion, particularly for foods high in protein and fat, and further studies with longer test sessions (3–5 h) are warranted. Additional testing of branded foods needs to be undertaken in order to develop a more comprehensive database of foods. This will be necessary if the FII is to be widely incorporated into clinical practice. This testing will also allow further exploration of the relationship between dietary factors and physiological insulin secretion evoked by foods and dietary factors.

## 5. Conclusions

In conclusion, the present study found wide variations in the observed insulin responses both within and between food groups. The FII could not be accurately calculated based on carbohydrate content alone or the full nutritional composition of the food, implying that *in vivo* testing is required. Correlations between the FII and different nutrients indicate that the postprandial insulin response is not the effect of a single nutrient but rather the final result of *interactions* between nutrients and the food matrix itself.

## Figures and Tables

**Table 1:** Percentage contribution and significance level for each variable selected in a stepwise multiple linear regression analysis to predict the Food Insulin Index (FII) in Equation (1).

Variable Predicting FII	Contribution (%)	*p* Value
Glucose Score	85.1	<0.0001
Sugar	6.9	<0.0001
Protein	6.7	<0.0001
Fat	1.3	0.053

## Supplementary Materials

The following are available online at http://www.mdpi.com/2072-6643/8/4/210/s1. Table S1: Macronutrient composition, glycaemic index (GI), glycaemic load (GL), actual glucose score (GS), and food insulin index (FII) for 1000-kJ portions of the reference glucose and test foods.

## Abbreviations

The following abbreviations are used in this manuscript:

FII	Food Insulin Index
GS	Glucose Score
GI	Glycemic Index
GL	Glycemic Load
iAUC	incremental Area Under the Curve
ISO	International Standard of Operation
