# Supplementary material for: Algorithms to Improve the Prediction of Postprandial Insulinaemia in Response to Common Foods
Source: Nutrients. 2016 Apr 8;8(4):210. doi: 10.3390/nu8040210 (PMC4848679; doi:10.3390/nu8040210)
Supplement: Supplementary file 1 [file nutrients-08-00210-s001.docx]

Supplementary Materials: Algorithms to Improve the Prediction of Postprandial Insulinaemia in Response to Common Foods

Kirstine J. Bell, Peter Petocz, Stephen Colagiuri and Jennie C. Brand-Miler

**Table S1.** Macronutrient composition, glycaemic index (GI), glycaemic load (GL), actual glucose score (GS), and food insulin index (FII) for 1000-kJ portions of the reference glucose and test foods.

| **Food** | **Test Date** | **Weight (g/MJ)** | **Protein (g/MJ)** | | **Fat (g/MJ)** | | **AvCHO (g/MJ)** | | **Sugar (g/MJ)** | | **Fibre (g/MJ)** | | **GI (%)** | | **GL (g/MJ)** | | **GS (%)** | | | **FII (%)** |
| --- | --- | --- | --- | --- | --- | --- | --- | --- | --- | --- | --- | --- | --- | --- | --- | --- | --- | --- | --- | --- |
| **Vegetables and legumes** |  |  |  | |  | |  | |  | |  | |  | |  | |  | | |  |
| Peas, steamed from frozen (McCains) * | 2011 | 333 | 17 | | 1 | | 21 | | 7 | | 22 | | 22 | | 5 | | 40 ± 10 | | | 37 ± 8 |
| Carrots, peeled and steamed (Australia) * | 2011 | 775 | 9 | | 2 | | 32 | | 29 | | 33 | | 33 | | 14 | | 35 ± 5 | | | 44 ± 7 |
| Broccoli, steamed (Australia) * | 2011 | 877 | 4 | | 4 | | 11 | | 11 | | 25 | | - | | - | | 16 ± 5 | | | 29 ± 8 |
| Cauliflower, steamed (Australia) * | 2011 | 971 | 21 | | 2 | | 19 | | 19 | | 27 | | - | | - | | 31 ± 7 | | | 48 ± 9 |
| Sweet Potato, orange, peeled and steamed (Australia) * | 2011 | 313 | 6 | | 0 | | 48 | | 19 | | 10 | | 61 | | 29 | | 100 ± 11 | | | 96 ± 14 |
| Butternut pumpkin, baked (Australia) * | 2011 | 431 | 3 | | 11 | | 37 | | 28 | | 21 | | 51 | | 19 | | 64 ± 15 | | | 77 ± 16 |
| 4 Bean Mix (Edgells) | 2013 | 201 | 16 | | 6 | | 33 | | 5 | | 16 | | 37 | | 12 | | 31 ± 12 | | | 34 ± 14 |
| **Fruits** |  |  |  | |  | |  | |  | |  | |  | |  | |  | | |  |
| Peach, raw (Australia) | 2011 | 565 | 7 | | 1 | | 51 | | 46 | | 13 | | 56 | | 29 | | 53 ± 17 | | | 39 ± 18 |
| **Dairy products** |  |  |  | |  | |  | |  | |  | |  | |  | |  | | |  |
| Chocolate milk (Moove) | 2013 | 341 | 19 | | 6 | | 35 | | 34 | | 0 | | 26 | | 9 | | 46 ± 17 | | | 46 ± 23 |
| Yoghurt, plain (Dairy Farmers) | 2013 | 213 | 10 | | 7 | | 34 | | 33 | | 0 | | 18 | | 6 | | 22 ± 14 | | | 46 ± 19 |
| Brie Cheese (Coles) | 2013 | 65 | 11 | | 21 | | 1 | | 1 | | 0 | | - | | - | | 3 ± 5 | | | 7 ± 5 |
| Custard (Dairy Farmers) | 2013 | 233 | 10 | | 6 | | 36 | | 32 | | 0 | | 29 | | 10 | | 32 ± 10 | | | 57 ± 36 |
| Cream (Dairy Farmers) | 2013 | 72 | 2 | | 25 | | 2 | | 2 | | 0 | | - | | - | | 1 ± 1 | | | 8 ± 8 |
| **Protein foods** |  |  | |  | |  | |  | |  | |  | |  | |  | |  | | |
| Lamb, grilled | 2013 | 136 | | 38 | | 10 | | 0 | | 0 | | 0 | | - | | - | | 6 ± 4 | 41 ± 19 | |
| Pork, grilled | 2013 | 239 | | 53 | | 3 | | 0 | | 0 | | 0 | | - | | - | | 3 ± 3 | 19 ± 10 | |
| Ham, shaved (Coles) | 2013 | 213 | | 34 | | 11 | | 0 | | 0 | | 0 | | - | | - | | 10 ± 6 | 19 ± 11 | |
| Beef sausage, thin, grilled (Coles) | 2013 | 106 | | 14 | | 19 | | 35 | | 0 | | 0 | | - | | - | | 5 ± 5 | 7 ± 9 | |
| Beef meat pie (Four’n’Twenty) | 2013 | 104 | | 10 | | 12 | | 23 | | 0 | | - | | - | | - | | 31 ± 10 | 41 ± 19 | |
| Chicken nuggets (Ingham) | 2013 | 99 | | 18 | | 13 | | 18 | | 1 | | - | | 46 | | 8 | | 29 ± 8 | 41 ± 26 | |
| Sushi, chicken roll (I Love Sushi) | 2013 | 160 | | 19 | | 4 | | 37 | | 5 | | - | | 48 | | 18 | | 59 ± 12 | 48 ± 23 | |
| **Carbohydrate foods** |  |  | |  | |  | |  | |  | |  | |  | |  | |  |  | |
| Weetbix (Sanitarium) | 2013 | 67 | | 8 | | 1 | | 45 | | 2 | | 7 | | 69 | | 31 | | 48 ± 19 | 41 ± 16 | |
| Plain Biscuit, Arrowroot (Arnotts) | 2013 | 54 | | 3 | | 6 | | 42 | | 12 | | 0 | | 69 | | 29 | | 58 ± 14 | 48 ± 20 | |
| Tim Tam (Arnotts) | 2013 | 46 | | 2 | | 12 | | 30 | | 20 | | 0 | | - | | - | | 32 ± 11 | 27 ± 15 | |
| Hokkien Noodles (Kan Tong) | 2013 | 149 | | 10 | | 1 | | 47 | | 0 | | 0 | | - | | - | | 43 ± 25 | 22 ± 9 | |
| Couscous (San Remo) | 2013 | 67 | | 8 | | 1 | | 48 | | 0 | | 2 | | 65 | | 31 | | 66 ± 23 | 84 ± 43 | |
| Butter chicken sauce (Sharwood’s) | 2013 | 209 | | 3 | | 16 | | 21 | | 9 | | 0 | | - | | - | | 22 ± 9 | 16 ± 7 | |

Mean ± SEM. * Tested in 300 kJ portion and GS and FII determined against 300 kJ Glucose.
